# Emerging Functions of Nano-Organized Polysaccharides

**DOI:** 10.3390/nano12081277

**Published:** 2022-04-08

**Authors:** Takuya Kitaoka

**Affiliations:** Department of Agro-Environmental Sciences, Graduate School of Bioresource and Bioenvironmental Sciences, Kyushu University, Fukuoka 819-0395, Japan; tkitaoka@agr.kyushu-u.ac.jp; Tel.: +81-92-802-4665

Natural polysaccharides, such as cellulose and chitin, possess unique hierarchical nanoarchitectures, e.g., crystalline, fibrous, and needle-like structures, in which each macromolecular component assembles in a regular and organized manner during biosynthesis and/or physicochemical processing. Among the various nano-organized polysaccharides, nanocellulose obtained from plants and bacteria is the most promising natural nanomaterial in practical applications due to its high aspect ratio, high elastic modulus, high transparency, low thermal expansion coefficient, and other fascinating properties. Chitin nanofibers from crabs and shrimps are also expected to produce advanced nanomaterials for cosmetics and biomedical applications. Starch, xylan, and other polysaccharides have recently been studied for novel applications as nanomaterials. Research on and development of natural polysaccharides are classified into two categories: (1) greener alternatives to existing products from ecological and sustainability viewpoints and (2) emerging functional nanomaterials from scientific encounters with the unknown. Both approaches are important for advancing the utilization of natural polysaccharides.

The aim of this Special Issue, entitled *Emerging Functions of Nano-Organized Polysaccharides*, is to showcase the current challenges involved with new conceptual and functional designs of nano-polysaccharide materials for a diverse range of future applications. The unexpected new functions arising from the inherent nanoarchitectures of natural nano-organized polysaccharides will provide new insights into polysaccharide-driven nanomaterial chemistry and engineering. This Special Issue comprises one review paper and ten research articles. The combination of various natural polysaccharides and unique strategies for functional material design will open up new avenues for the emerging functions of natural nano-polysaccharides.

First of all, the review article provided by Miyagi and Teramoto comprehensively covers the recent advances in the construction of functional materials in various material forms from cellulosic cholesteric liquid crystals [1]. The spontaneous and controllable self-assembling features of cellulosic nanomaterials are the most fascinating phenomenon for materials design, and this review provides information on both the fundamentals and the structure-driven functions of cellulosic cholesteric nanomaterials in various forms.

Strong, tough, and high-performance nanocellulose composites are typical materials that draw public attention. Sakuma et al. successfully fabricated millimeter-thick, transparent cellulose nanofiber (CNF)/polymer composites using CNF xerogels with high porosity as a template [2]. The as-designed composites demonstrate high flexural modulus, low thermal expansion, and good flame-retardant properties. Beaumont et al. highlight the solvent-free sol/gel process for the facile preparation of mechanically robust CNF/silica nanocomposites in combination with methylcellulose and starch [3]. Strong and shapable 3D CNF/silica hydrogels can act as adsorbers for heavy metals and dyes. Zhu et al., developed 3D nanocarbon materials via the polydopamine doping and pyrolysis of CNF paper [4]. This work achieved a high yield of 3D porous nanocarbons with good specific capacitance up to 200 F g^−1^. Tsuneyasu et al. exhibit the successful enhancement of luminance in powder electroluminescent devices using smooth and transparent CNF films [5]. The optical and morphological properties of CNF substrates make a great contribution to increasing both the current density and luminance of the devices. Fukuda et al. introduce the enzymatic preparation of spherical microparticles from artificial lignin and CNF for cosmetic applications [6]. Lignin is also an abundant, renewable, and biodegradable biopolymer, comparable to natural polysaccharides, and the combination of lignin and CNF will attract much attention in a carbon-neutral eco-society. Studies like those can expand the possibility of creating high-value nanomaterials with nano-polysaccharides like CNF.

Apart from nanocellulose, other polysaccharides are also promising for the design of advanced functional materials. Xylan is a highly abundant, plant-based form of biomass and is expected to find further uses. Wang and Xiang successfully prepared a highly stable Pickering emulsion with xylan hydrate nanocrystals with a uniform size [7]. This is a pioneering work in the use of xylan-derived nanomaterials as a solid surfactant emulsifier. Chitin is the second most abundant polysaccharide after cellulose and has unique hierarchical nanoarchitectures similar to those of nanocellulose. Wang et al. report the thermal conductivity of chitin and deacetylated-chitin nanofiber films and reveal the relationship between the in-plane thermal conductivity and surface chemistry [8]. A computational and theoretical study on non-covalent interactions with nano-polysaccharides is of great significance to advancing the materials science of natural biomaterials. Yui et al. provide computational predictions for the molecular and crystal structures of a chitosan–zinc chloride complex [9]. Chitosan chains of antiparallel polarity form zigzag-shaped chain sheets, and the coordination of Zn ions with chitosan has been proposed. In the days ahead, the digital transformation of materials science will become more and more important for the unexpected discovery of unknown functions of nano-organized polysaccharides.

The biomedical applications of natural nano-polysaccharides have recently aroused great interest in tissue engineering and various therapeutics. Noda et al. report on the unique combination of surface-carboxylated nanocellulose and surface-deacetylated chitin nanofibers to promote the adhesion, migration, and proliferation of fibroblast cells [10]. Bioinert CNF can be designed to prepare bioadaptive materials by surface carboxylation, and cellular migration can be controlled using special nano-polysaccharides with a core–shell structure of chitin and chitosan, respectively. Moreover, bacterial nanocellulose is also a fascinating nanomaterial, as well as plant-derived nanocellulose. Ando et al. emphasize the feasibility of nanofibrillated bacterial cellulose as a carrier of doxorubicin to promote therapeutic outcomes in gastric cancer [11]. Bacterial cellulose is very effective for the drug loading and release of various anticancer agents, making it a promising nanomaterial for advanced drug delivery systems in cancer therapy.

In summary, pioneering work on the extraordinary and emerging functions of nano-organized polysaccharides, such as nanocellulose, chitin/chitosan nanofibers, and other nano-polysaccharides, will signal a new trend in the research and development of natural nanomaterials as we move towards achieving the UN’s Sustainable Development Goals. All the papers published in this Special Issue will present readers with new concepts and ideas for the next-generation material design of nano-organized polysaccharides. Last but certainly not least, I would like to express my great appreciation to all the authors for their excellent work, the reviewers for their constructive contributions, and the editorial board members for their support throughout the process of setting up this Special Issue.

## References

[1] Miyagi K., Teramoto Y. (2021). Construction of Functional Materials in Various Material Forms from Cellulosic Cholesteric Liquid Crystals. Nanomaterials.

[2] Sakuma W., Fujisawa S., Berglund L.A., Saito T. (2021). Nanocellulose Xerogel as Template for Transparent, Thick, Flame-Retardant Polymer Nanocomposites. Nanomaterials.

[3] Beaumont M., Jahn E., Mautner A., Veigel S., Böhmdorfer S., Potthast A., Gindl-Altmutter W., Rosenau T. (2022). Facile Preparation of Mechanically Robust and Functional Silica/Cellulose Nanofiber Gels Reinforced with Soluble Polysaccharides. Nanomaterials.

[4] Zhu L., Uetani K., Nogi M., Koga H. (2021). Polydopamine Doping and Pyrolysis of Cellulose Nanofiber Paper for Fabrication of Three-Dimensional Nanocarbon with Improved Yield and Capacitive Performances. Nanomaterials.

[5] Tsuneyasu S., Watanabe R., Takeda N., Uetani K., Izakura S., Kasuya K., Takahashi K., Satoh T. (2021). Enhancement of Luminance in Powder Electroluminescent Devices by Substrates of Smooth and Transparent Cellulose Nanofiber Films. Nanomaterials.

[6] Fukuda N., Hatakeyama M., Kitaoka T. (2021). Enzymatic Preparation and Characterization of Spherical Microparticles Composed of Artificial Lignin and TEMPO-oxidized Cellulose Nanofiber. Nanomaterials.

[7] Wang S., Xiang Z. (2021). Highly Stable Pickering Emulsions with Xylan Hydrate Nanocrystals. Nanomaterials.

[8] Wang J., Kasuya K., Koga H., Nogi M., Uetani K. (2021). Thermal Conductivity Analysis of Chitin and Deacetylated-Chitin Nanofiber Films under Dry Conditions. Nanomaterials.

[9] Yui T., Uto T., Ogawa K. (2021). Molecular and Crystal Structure of a Chitosan–Zinc Chloride Complex. Nanomaterials.

[10] Noda T., Hatakeyama M., Kitaoka T. (2022). Combination of Polysaccharide Nanofibers Derived from Cellulose and Chitin Promotes the Adhesion, Migration and Proliferation of Mouse Fibroblast Cells. Nanomaterials.

[11] Ando H., Mochizuki T., Lila A.S.A., Akagi S., Tajima K., Fujita K., Shimizu T., Ishima Y., Matsushima T., Kusano T. (2021). Doxorubicin Embedded into Nanofibrillated Bacterial Cellulose (NFBC) Produces a Promising Therapeutic Outcome for Peritoneally Metastatic Gastric Cancer in Mice Models via Intraperitoneal Direct Injection. Nanomaterials.

